# Privacy Concerns Versus Personalized Health Content—Pregnant Individuals’ Willingness to Share Personal Health Information on Social Media: Survey Study

**DOI:** 10.2196/60862

**Published:** 2025-02-10

**Authors:** Haijing Hao, Yang W Lee, Marianne Sharko, Qilu Li, Yiye Zhang

**Affiliations:** 1 Bentley University, Boston, MA, United States; 2 Northeastern University, Boston, MA, United States; 3 Weill Cornell Medicine, 575 Lexington Ave, New York, NY, 10022, United States, 1 646-962-9437

**Keywords:** privacy concerns, trust, pregnancy, health information seeking, pregnant women, maternal, maternity, childbearing, web-based information, health information, mental health, internet, social support, technology, mobile health, mHealth, digital health, health informatics, social media

## Abstract

**Background:**

Often lacking immediate access to care providers, pregnant individuals frequently turn to web-based sources for information to address their evolving physical and mental health needs. Social media has gained increasing prominence as a source of news and information despite privacy concerns and unique risks posed to the pregnant population.

**Objectives:**

This study investigated the extent to which patients may be willing to disclose personal health information to social media companies in exchange for more personalized health content.

**Methods:**

We designed and deployed an electronic survey to pregnant individuals worldwide electronically in 2023. We used the classical Internet Users’ Information Privacy Concerns (IUIPC) model to examine how privacy concerns modulate pregnant individuals’ behaviors and beliefs regarding risk and trust when using social media for health purposes. Results were analyzed using partial least squares structural equation modeling.

**Results:**

Among 317 respondents who initiated the survey, 84% (265/317) of the respondents remained in the study, providing complete responses. Among them, 54.7% (145/265) indicated willingness to provide their personalized health information for receiving personalized health content via social media, while 26% (69/265) were uncertain and 19.3% (51/265) were opposed. Our estimated IUIPC model results are statistically significant and qualitatively align with the classic IUIPC model for the general population, which was previously found in an e-commerce context. The structural model revealed that privacy concerns (IUIPC) negatively affected trusting beliefs (β=−0.408; *P*<.001) and positively influenced risk beliefs (β=0.442; *P*<.001). Trusting beliefs negatively impacted risk beliefs (β=−o.362; *P*<.001) and positively affected the intention to disclose personal health information (β=o.266; *P*<.001). Risk beliefs negatively influenced the intention to disclose (β=−0.281; *P*<.001). The model explained 41.5% of the variance in the intention to disclose personal health information (*R*²=0.415). In parallel with pregnant individuals’ willingness to share, we find that they have heightened privacy concerns and their use of social media for information seeking is largely impacted by their trust in the platforms. This heightened concern significantly affects both their trusting beliefs, making them less inclined to trust social media companies, and their risk beliefs, leading them to perceive greater risks in sharing personal health information. However, within this population, an increase in trust toward social media companies leads to a more substantial decrease in perceived risks than what has been previously observed in the general population.

**Conclusions:**

We find that more than half of the pregnant individuals are open to sharing their personal health information to receive personalized content about health via social media, although they have more privacy concerns than the general population. This study emphasizes the need for policy regarding the protection of health data on social media for the pregnant population and beyond.

## Introduction

### Background

Advancements in web-based technology have significantly enhanced opportunities for digital social support and web-based health information seeking [1,2]. The trend of searching for health information on the web has gained momentum [3-5], particularly since the COVID-19 pandemic where health care services were overburdened to address individual questions [6,7]. Instead, patients gathered on the web, using social media in particular, for guidance and resources to empower themselves, cope with stress, and strengthen positive social support. This health information seeking behavior is particularly prevalent among pregnant patients [8]. Pregnancy is a vulnerable period that exposes patients to heightened anxiety, depression, and stress, leading to adverse maternal, infant, and family outcomes, disproportionately affecting disadvantaged families [9,10]. The negative impact can be mitigated by interventions from health care providers [11,12]. However, disparities in access to health care, health literacy, socioeconomic status, and neighborhood characteristics strangle equitable access to clinical interventions [12-14]. As a result, many patients resort to web-based information seeking and social support to relieve stressors and resolve individual needs during pregnancy [15]. In fact, people of childbearing age are one of the most active users in the digital space in the United States [16,17]. An estimated three-quarters of the US pregnant population seek pregnancy and birth information on the web, and there is increasing IT acceptance for data sharing [18,19]. Social media is an overwhelmingly popular source for pregnancy information, with myriad of social media accounts and influencers distributing a wide range of information and offering venues for discussion [8,20]. For example, research showed that web-based support and interventions improved health outcomes among pregnant patients with gestational diabetes mellitus, and individualized health interventions resulted in lower odds of developing gestational diabetes mellitus [21].

Yet, privacy and risk concerns are important to consider when using digital health technologies for pregnancy [22,23]. Research has shown that individuals often possess both risk and privacy concerns when turning to social media for health information [24-28]. For example, pregnancy privacy concerns have heightened since the overturn of Dobbs v Jackson by the Supreme Court in the United States, which had protected an individual’s right to abortion. This has resulted in variable US state laws criminalizing certain reproductive health care, amplifying privacy concerns for pregnant individuals seeking web-based information about abortions and storing reproductive health information on personal applications [29]. However, despite significant privacy concerns, given patients’ overwhelming health needs and the lack of clinical resources to address them, social media remains as a popular source for seeking health information and digital social support.

### Objectives

Given the increasing need for information and limited access to prompt responses from health care providers regarding pregnancy-related inquiries, there is a notable tension between privacy concerns and the demand for personalized health communication. Our study aims to fill a significant research gap by applying the Internet Users’ Information Privacy Concerns (IUIPC) model to understand pregnant individuals’ privacy concerns when seeking health information and social support on social media. While prior studies have used the IUIPC model to examine privacy issues across various digital technologies and populations, none have specifically focused on pregnant individuals—a group that is particularly active on the web and yet vulnerable due to heightened health needs and privacy risks. By investigating how privacy concerns, trusting beliefs, and risk perceptions influence pregnant individuals’ willingness to disclose personal health information for personalized content, our research contributes to the literature by providing new insights into the information disclosure behaviors of this unique population on social media. This enhances the understanding of privacy dynamics in digital health contexts and informs strategies for health care providers, policy makers, and social media companies to better support pregnant individuals’ needs.

## Methods

### Study Design

This is a survey study. We developed and deployed a survey on Prolific in March 2023. Prolific is a web-based platform that connects researchers with a pool of prescreened study participants globally. Earlier studies have shown that participants recruited from Prolific provide high-quality data regarding users’ perceptions of software and digital platforms [30,31]. The survey questions were hosted on Qualtrics (Qualtrics), a survey software. The survey comprises 41 questions: 22 questions focus on the conceptual model, with 7-point Likert scales, and the remaining questions focus on demographic information, social media questions related to health, and 3 quality-check questions. The survey questions are listed in Table S1 in Multimedia Appendix 1.

### Settings

Participants were recruited using Prolific’s recruitment method based on inclusion criteria: (1) aged at least 18 years or older, and (2) currently or recently pregnant within 2 years prior to the time of the survey. We used Prolific’s prescreening filters to identify eligible participants, including age, gender, and pregnancy status to align with our inclusion criteria. Eligible participants identified through the prescreening process received an invitation to participate in our study via Prolific’s messaging system.

The invitation included a brief overview of the study’s purpose, estimated time commitment, and the compensation offered. Participants were excluded if their responses in the survey were incomplete or of poor quality, as determined by the quality-check questions. The first quality check is based on age, using the following questions: Q29_1 “What is your age?—Age” and Q14_1 “What is your birth year?—Year,” which were placed in 2 different sections of the survey. If the difference between the answers to these 2 questions is more than 2 years, we excluded that participant. The second quality check is based on the Collection construct from the IUIPC model, using the following questions: Q18_1 “It usually bothers me when health-conscious social media companies ask me for personal health information.” and Q18_5 “It usually doesn’t bother me when health-conscious social media companies ask me for personal health information.” These 2 opposite questions were placed in the survey, and if participants provide similar responses to both (eg, strongly disagree or strongly agree for both), they were excluded. However, if both answers are neutral (neither agree nor disagree), the participants were retained. The third quality check is based on the Risk-Belief construct from the IUIPC model, using the following questions: Q20_1 “In general, it would be risky to give my health information to health-conscious social media companies.” and Q20_5 “I would feel safe giving my health information to health-conscious social media companies.” The same strategy as the second quality check applies to this pair of questions.

### Ethical Considerations

Our study was approved as exempt under institutional review board category II by Bentley University (no. 2023011). Upon accessing the survey link, participants were presented with an informed consent form detailing the study’s objectives, procedures, potential risks, benefits, and the voluntary nature of participation. Participants had to electronically agree to the consent form before proceeding to the survey questions. The study data are fully anonymous. Each participant received a US $1 reward for answering the questions per the site’s rules. Compensation was managed through Prolific’s system to ensure timely and secure payment.

### Variables and Data Sources

The survey draws on a conceptual model, the IUIPC model by Malhotra et al [32]. IUIPC model was designed to evaluate whether concerns about information privacy sway an individual’s intent to adopt digital technology in the e-commerce context [32]. Since then, numerous studies have delved into various populations’ privacy concerns across various digital technologies based on the IUIPC model [26,33-38]. However, limited knowledge exists on the perspective of pregnant individuals’ privacy concerns when seeking health information on social media. Thus, we aim to uncover how information privacy concerns may influence pregnant individuals’ intentions to reveal personal health data to receive personalized pregnant-related health content through social media using IUIPC.

**Figure 1.**
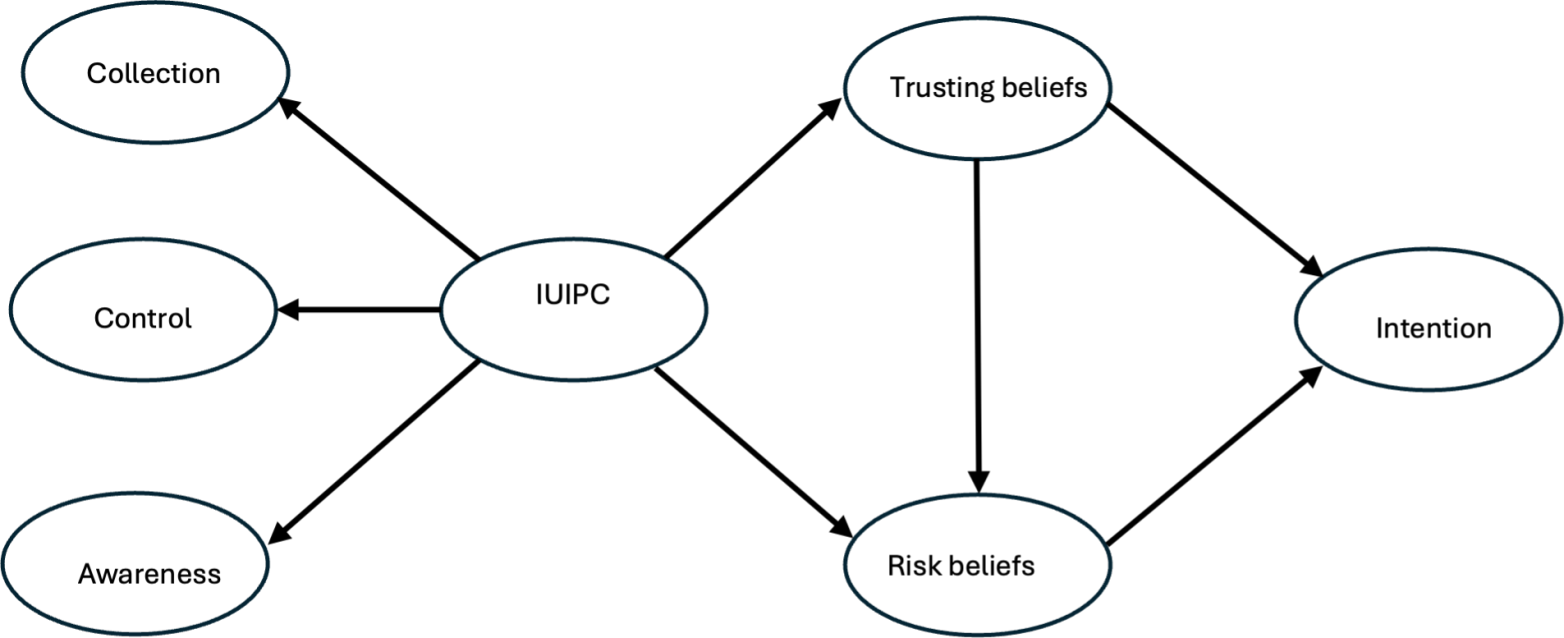
Internet Users’ Information Privacy Concerns (IUIPC) with 5 major constructs: collection, control, awareness, trusting beliefs, and risk beliefs.

Figure 1 depicts the structure of the basic theoretical IUIPC model, consisting of 5 major constructs: collection, control, awareness, trusting beliefs, and risk beliefs [32]. Collection, control, and awareness are 3 first-order constructs, which are directly measured through survey items. IUIPC is a second-order construct, or a latent construct, which is estimated by the 3 first-order constructs. In this study, the collection construct refers to the extent of an individual’s privacy concerns about whether it is worth revealing their personal information to social media companies. The control construct measures the degree to which individuals feel they have control over their personal data and know how the data will be collected, used, and shared. The awareness construct gauges the level of a user’s privacy concern regarding their awareness of social media companies’ transparency and the proper handling of their information. Trusting beliefs refer to the degree to which people believe an entity is trustworthy in protecting their personal information, whereas risk beliefs refer to the expectation of a potential loss when sharing personal information with the entity. In this study, the personal information is personal health information and the entity is social media companies. IUIPC integrates trusting beliefs and risk beliefs to elucidate why a user may have an intention to adopt a new technology for certain benefits. In the context of this study, IUIPC reflects social media users’ concerns about social media companies’ collection of personal information, the users’ control over the collected information, and the users’ awareness of how the collected information is used, which in turn affects users’ intentions to reveal personal health information to receive customized health information through social media.

### Research Hypotheses

Previous research has demonstrated that users with greater privacy concerns regarding social media tend to have lower trust toward these platforms [36,38]. Therefore, we hypothesize the following:

- Hypothesis 1: For pregnant individuals, IUIPC will have a negative effect on their trusting beliefs.

There is often an inverse relationship between trusting beliefs and risk beliefs because trust implies a firm belief in the reliability of something, while risk indicates the possibility of negative outcomes. When users exhibit higher privacy concerns toward social media, it implies that they perceive a greater risk associated with its use [36,38]. Thus, we propose the following 2 hypotheses:

- Hypothesis 2: For pregnant individuals, IUIPC will have a positive effect on their risk beliefs.
- Hypothesis 3: For pregnant individuals, their trusting beliefs will have a negative effect on their risk beliefs.

Research has shown that trust in a specific technology positively influences individuals’ intention to explore that technology [39]. Pregnant individuals will share their personal information in exchange for health information through social media if they trust the companies and find the specific health content valuable enough to justify the privacy trade-off. Previous studies have also shown that trust plays a crucial role in adopting social media [33,34,40]. Therefore, we further hypothesize the following:

- Hypothesis 4: For pregnant individuals, trusting beliefs have a positive effect on their intention to reveal personal health information to social media companies to receive personalized health content through social media.

A previous study on risk beliefs concerning social media suggested that a perception of risk by users made them less likely to adopt social media [38]. If pregnant individuals perceive that sharing personal health information with social media companies for health purposes is risky, then this perception will result in a lower intention to use. Thus, we hypothesize the following:

- Hypothesis 5: For pregnant individuals, risk beliefs have a negative effect on their intention to reveal personal health information to social media companies in exchange for personalized health content through social media.

### Measurement Model and Structural Equation Model Evaluation

We used partial least squares structural equation modeling to estimate the IUIPC model and test our hypotheses. Partial least squares structural equation modeling is a variance-based structural equation modeling (SEM) technique suitable for exploratory research and complex models with latent constructs, particularly when the sample size is small and data that may not meet the strict assumptions of covariance-based SEM [41]. We estimated the IUIPC model and assessed the measurement model results according to the following 4 criteria: unidimensionality, item reliability, construct reliability, and convergent validity [39,40]. We used Stata SE 17, and the Stata add-on package, plssem (StataCorp) [42] for the analysis. We reported the *P* values associated with each path coefficient in the structural model to indicate the statistical significance of the hypothesized relationships with significance at the .05 level (*P*<.05). CIs were determined using bootstrapping with 5000 resamples. Dimensionality is the number of constructs that a group of items or a group of survey questions reflects in a formative measurement model. We conducted principal component factor analysis to examine eigenvalues and kept only the constructs whose eigenvalues are >1. Item reliability refers to the extent to which an observed item can reliably measure its corresponding construct. We assume reliability when the standardized loading of an item on their constructs is >0.7. Construct reliability refers to the degree to which the items of a construct consistently and accurately measure that construct. Two commonly used metrics to evaluate construct reliability are Cronbach α and Dillon-Goldstein ρ, and their generally acceptable values are >0.7. Convergent validity evaluates the extent to which a set of items designed to measure the same construct are strongly related to one another. Average variance extracted (AVE) measures the average amount of variance in the items explained by the construct. AVE values should be >0.5, meaning that the construct explains 50% or more of the variance in its indicators. We used the *R*^2^ value and the goodness of fit to evaluate the predictive power of IUIPC, which is a structural model. The acceptable threshold for goodness of fit is 0.36 [43]. Finally, variance inflation factor values are used to check for multicollinearity, with a common threshold of 2.5.

## Results

### Descriptive Statistics

The data collection lasted for 2 days until we reached the goal of 300 responses. A total of 317 respondents answered the survey. After removing incomplete surveys and responses that did not meet our quality criteria based on the 3 quality check questions, 83.6% (265/317) of the respondents remained in the study. Table 1 shows the sociodemographic information of the respondents. The largest racial group is White (164/265, 61.9%), and the second largest racial group is African American (82/265, 30.9%). Nearly 75% (198/265) of the respondents have a bachelor’s degree or above, which matches our expectations and is consistent with previous studies showing that participants on Prolific tend to have higher levels of education than the general population [44].

Most participants (256/265, 96.7%) responded that they receive their pregnancy health information from their doctors, and 69.4% (184/265) of the participants mentioned the web or social media as pregnancy health information sources. Regarding specific social media platforms, 74.7% (198/265) used Instagram, 69.4% (184/265) used Facebook, 55.5% (147/265) used TikTok, and 30.1%(81/265) used Twitter (Now X). A small percentage (9/265, 3.4%) responded that they used PatientsLikeMe, a health-focused social media platform. An additional 4.2% (11/265) named other social media platforms including Reddit, LinkedIn, YouTube, Pinterest, and WhatsApp. Finally, 0.74% (2/265) of participants did not use social media. Overall, 99.2% (263/265) of respondents used 1 or more social media platforms. Among all 265 participants, 54.7% (145/265) answered that they were comfortable sharing their personal health information in order to receive customized health content on social media, whereas 26% (69/265) were uncertain and 19.3% (51/265) responded that they were not comfortable sharing.

**Table 1.** Descriptive statistics of the 265 participants who were enrolled in the survey study.

Participant characteristics		Values, n (%)
Race		
	Asian	8 (3)
	African American	82 (30.9)
	Multirace	5 (1.9)
	White	164 (61.9)
	Other	6 (2.3)
Ethnicity		
	Hispanic	14 (5.3)
	Non-Hispanic	228 (86)
	Prefer not to say	23 (8.7)
Marital status		
	Married	126 (47.6)
	In a relationship	111 (41.9)
	Single^a^	28 (10.6)
Education		
	High school	23 (8.7)
	Some college	44 (16.6)
	Bachelor’s degree	141 (53.2)
	Master’s degree	47 (17.7)
	Doctoral degree	2 (0.8)
	Professional degree	8 (3)

a Single includes separated, divorced, widowed, and single.

### Outcome Data

Of those who were willing to share their personal health information to receive personalized health content, all of them used 1 or more popular social media platforms such as Facebook, Instagram, TikTok, or Twitter. Of those who responded that they were not comfortable sharing, 47% (24/51) cited privacy concerns, and equally, 47% (24/51) cited a lack of trust in social media companies and influencers. One respondent commented on the political environment as a concern for sharing. There were no significant differences in ethnicity, race, or education level (bachelor’s degree or more vs up to high school) in the responses. Among users of the top four social media platforms, 56.1% (111/198) of Instagram users, 56.5% (104/184) of Facebook users, 64% (94/147) of TikTok users, and 65.4% (53/81) of Twitter (now X) users were comfortable sharing personal health information in exchange for more customized social media content. There was a significant difference in willingness between people who use and do not use TikTok, and between those who do and do not use Twitter (now X).

### Measurement Model Result

**Figure 2.**
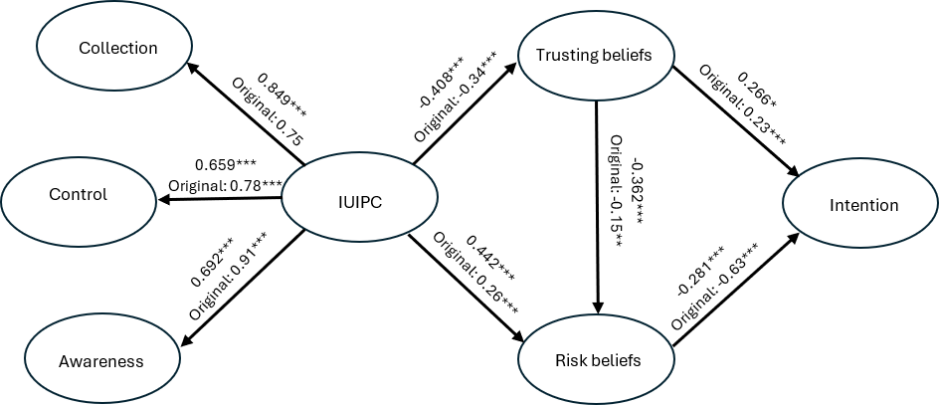
Internet Users’ Information Privacy Concerns (IUIPC) model with estimated path coefficients all statistically significant and are in the directions we expected, confirming that those items are meaningful for our model. The asterisks denote statistical significance based on *P* values. *P<.05, **P<.01. ***P<.001

Overall, the measurement model results demonstrate that our constructs exhibit strong reliability and validity, confirming their appropriateness for use in the structural analysis. The collection, control, and awareness constructs all had eigenvalues >1. Thus, they were kept in the model as unidimensional constructs. The loadings and constructs’ AVE values are shown in Tables S2 and S3 in Multimedia Appendix 1. As Table S2 in Multimedia Appendix 1 shows, most of the bolded diagonal block loadings are >0.7 for the corresponding constructs, which demonstrate the items’ strong reliability. Although a few items on the second-order construct, IUIPC, are slightly <0.7, we still accept this model result because these loadings are not trivial, ranging from 0.44 to 0.58. In addition, they are still statistically significant. Furthermore, as Figure 2 shows, among the estimated coefficients for the structural model, all the path coefficients are statistically significant and are in the directions we expected, which confirms that those items are meaningful for our model. In Table S2 in Multimedia Appendix 1, we can see that the 2 commonly used metrics for evaluating construct reliability, Cronbach α and Dillon-Goldstein ρ, are >0.7, above the acceptable threshold value, indicating good construct reliability of our model. As Table S3 in Multimedia Appendix 1 shows, all constructs’ AVE values are >0.5 except for the IUIPC, the second order construct. This AVE value is <0.5 because of the lower loadings of the control and awareness items on IUIPC, as shown in Table S2 in Multimedia Appendix 1. Finally, the model’s composite reliability scores, as measured by Dillon-Goldstein ρ of all constructs (Table S2 in Multimedia Appendix 1), are >0.7, indicating good internal consistency of the IUIPC model.

### Structural Model Result

Our estimated IUIPC model results and the 5 path coefficients of the structural model support all 5 hypotheses that we constructed. The IUIPC model’s average *R*^2^ value is 0.4146, indicating a moderate effect. The absolute goodness of fit is 0.4923, which is >0.36, the acceptable threshold [43]. All variance inflation factor values were <2.5, the commonly suggested threshold value (Table S4 in Multimedia Appendix 1), indicating little concern for multicollinearity in our structural model. As Figure 2 shows, all path coefficients are statistically significant and in the directions as we expected, and most of them are qualitatively consistent with the original IUIPC model estimates from the study by Malhotra et al [32].

## Discussion

### Interpretation

Our survey of 265 pregnant individuals globally finds that more than half of pregnant individuals are open to sharing their personal health information to receive personalized content about health via social media, although they have more privacy concerns than the general population. In the original study by Malhotra et al [32], the coefficient between the collection construct and the IUIPC was not statistically significant (β=0.75). However, we observed significance in this coefficient in our model (β=0.849; *P*<.001). This finding was supported by another study by Zeng et al [38], which examined the privacy concerns on social media platforms among the general population and found this coefficient to be at a similar significant level to ours (β=0.36; *P*<.001), albeit smaller. This may indicate that pregnant individuals are more careful about sharing their health-related information with social media companies, reflecting their privacy concerns. In this study, the loadings for both the control and awareness constructs are slightly lower than those in the original study by Malhotra et al [32]. Both our study and the study by Zeng et al [38] have loadings of control and awareness that are lower than results from the classic model by Maholtra et al [32]. This potentially indicates that today, social media users’ information control and social media users’ awareness of the social media company’s practices have a strong impact on their IUIPC concerns, although their quantitative effects are lower than those previously found in the e-commerce setting.

Our model results suggest that the impact of trusting beliefs on pregnant individuals’ intention to adopt social media for health purposes is greater than that found in the study by Malhotra et al [32]. The IUIPC in our model negatively affects trusting beliefs, evidenced by a path coefficient of β=−0.408 (*P*<.001), which is consistent with but stronger than the original model’s path coefficient of β=−0.34 (*P*<.001). This difference may suggest that pregnant individuals’ privacy concerns regarding trust are more pronounced than those found in the classical model [32]. The IUIPC construct in our model positively influences risk beliefs, with a coefficient of β=0.442 (*P*<.001), which is larger than the coefficient in the original model (β=0.26; *P*<.001). This suggests that, in addition to trust, pregnant individuals’ privacy concerns also have a stronger influence on their risk beliefs than those of the general population in the e-commerce context. In our model, trusting beliefs negatively impact risk beliefs, with a coefficient of β=−0.362 (*P*<.001), which is stronger than the original model’s coefficient of β=−0.15 (*P*<.001). This suggests that if a pregnant individual’s trust in a social media company increases, their perceived risks will decrease more than in the general population in the e-commerce context as previously found [32]. Trusting beliefs positively influence pregnant individuals’ intention to reveal health information to receive customized health content through social media with a coefficient of β=0.266 (*P*<.001), which is larger than the original model (β=0.23; *P*<.001). Conversely, risk beliefs have a negative effect on this intention with a coefficient of β=−0.281 (*P*<.001), which is smaller in magnitude than the original model (β=−0.63; *P*<.001). This is consistent with a previous study, which showed that trust is positively associated with adoption intention while privacy concerns are negatively associated with the adoption intention of technology in health care [45].

Our study adds to our current understanding that pregnant individuals are increasingly turning to social media for health information and support, despite significant privacy and risk concerns. For health care providers, this underscores the importance of integrating digital tools into their practice to meet patients where they are seeking information. By establishing a trustworthy web-based presence and providing accurate, evidence-based health information through social media, providers can better address the informational needs of pregnant patients. Educating patients about the potential risks associated with sharing personal health information on the web is also crucial. This includes guidance on maintaining privacy, recognizing credible sources, and understanding how personal data might be used by third parties. Enhancing the patient-provider relationship through open communication about privacy concerns can reduce the likelihood of patients seeking information from less reliable sources. For policy makers, our findings confirm a pressing need to safeguard personal health information on the web and in social media. Developing robust regulatory frameworks that govern how social media companies collect, use, and share personal health data is essential. Updating and enforcing privacy laws to encompass social media platforms can provide additional protections for sensitive health information. Policy makers can also educate individuals about digital privacy, helping pregnant individuals make informed decisions about sharing personal health information on the web.

Our results suggest that pregnant individuals are concerned about their personal health data being collected by social media companies, about losing control of their health information collected by social media companies, and about how their health information will be used. Privacy and confidentiality are paramount, as pregnant individuals may inadvertently disclose sensitive information that could be misused and harm them. Therefore, prior to collecting personal health information, social media companies with health-related content should explain clearly that the purpose of data collection is to create personalized health content for each user’s benefit. Furthermore, social media companies should guarantee that patients can easily access any information they have provided to ensure users’ rights to control their data. Users should also be informed, in clear language, of the companies’ privacy policies, including if and how their health data will be used. This transparency and control over their data may help build trust between pregnant individuals and the social media companies.

### Limitations

This study has limitations. First, since we distributed the survey to participants globally, various cultural differences, religions, and government policies may influence the perceptions of the participants. Second, our sample size was small and limited to web-based survey participants, which may have introduced a built-in bias toward the digital space. Our sample, recruited from Prolific, likely comprises individuals who are more digitally savvy compared with the general population, as reflected in their higher education levels and potentially greater web-based literacy, which may introduce selection bias and affect the generalizability of our findings. Future studies will recruit larger samples while specifically target participants with low web-based health literacy to ensure adequate representation of their perspectives and investigate whether there is any difference in the types of personal health data that participants are willing to share. Relatedly, while we did ask each participant which social media platforms they use, our small sample size did not allow for extensive platform-specific comparisons. In addition, our survey did not specify any social media or web-based health forum specifically but referred to general social media for health. Considering that privacy policies of various health-related social media platforms differ, users’ trusting beliefs and risk beliefs may differ across social media platforms. Furthermore, we acknowledge while our SEM allows for the testing of directional hypotheses, causality cannot be definitively established due to the cross-sectional nature of the data. Future studies should investigate additional factors such as sociodemographic moderating effects that may influence pregnant individuals’ intentions to use social media or web-based health platforms for health information seeking and communication. Moreover, future studies may investigate the benefit or cost of health information social media despite privacy concerns and consider extending our study by exploring how other vulnerable populations such as patients with chronic illnesses or mental health conditions navigate privacy and trust issues in the digital space. Finally, future research can consider designing and developing customized constructs to add to the classical IUIPC model. This would allow for examining how these tailored constructs impact pregnant patients’ privacy concerns on social media.

### Conclusions

In this study, we examined pregnant individuals’ willingness to disclose personal health information in exchange for more personalized health content on social media. Based on a classical model of IUIPC, we designed and deployed a survey for pregnant patients on the web. As found in previous studies such as those by Malhotra et al [32] and Zeng et al [38], our findings align with this consensus that higher privacy concerns negatively impact trust and positively influence risk perceptions in digital contexts. We found that pregnant individuals exhibit more pronounced social privacy concerns than those identified in the classic IUIPC model. This heightened concern significantly affects both their trusting beliefs, making them less inclined to trust social media companies, and their risk beliefs, leading them to perceive greater risks in sharing personal health information. However, within this population, an increase in trust toward social media companies leads to a more substantial decrease in perceived risks than what has been previously observed in the general population. Thus, social media companies delivering personalized-related health content should prioritize building trust with their users. This involves clearly informing patients about what health data are collected, providing them with easy access to their own health data, keeping them informed about how their health data are used, and including information about privacy protection policies. Given pregnant individuals’ needs for web-based health information seeking and the level of openness toward information sharing found from this study, we argue that more regulations are needed to mitigate users’ privacy and risk concerns and protect their data against exploitation on social media.

## Supplementary material

10.2196/60862 Multimedia Appendix 1 Supplementary tables.

## Abbreviations

AVE: average variance extracted
IUIPC: Internet Users’ Information Privacy Concerns
SEM: structural equation modeling
