# Supplementary material for: Privacy Concerns Versus Personalized Health Content—Pregnant Individuals’ Willingness to Share Personal Health Information on Social Media: Survey Study
Source: JMIR Form Res. 2025 Feb 10;9:e60862. doi: 10.2196/60862 (PMC11833185; doi:10.2196/60862)
Supplement: Multimedia Appendix 1 [file formative-v9-e60862-s001.docx]

# Appendix

**Table S1.** Survey Questions and Constructs

| Construct | Question | Item |
| --- | --- | --- |
| Collection | It usually bothers me when health-conscious social media companies ask me for personal health information. | Collection 1 |
|  | When health-conscious social media companies ask me for personal health information, I sometimes think twice before providing it. | Collection 2 |
|  | It bothers me to give personal health information to so many health-conscious social media companies. | Collection 3 |
|  | I’m concerned that health-conscious social media companies are collecting too much personal health information about me. | Collection 4 |
| Control | User’s social media privacy is really a matter of users’ right to exercise control and autonomy over decisions about how their information is collected, used, and shared. | Control 1 |
|  | User’s social media control of personal information is key to user privacy. | Control 2 |
|  | I believe that social media privacy is invaded when control is lost or unwillingly reduced as a result of a marketing transaction. | Control 3 |
| Awareness | Health-conscious social media companies seeking information online should disclose the way the data are collected, processed, and used. | Awareness 1 |
|  | A good social media privacy policy should have a clear and conspicuous disclosure. | Awareness 2 |
|  | It is very important to me that I am aware and knowledgeable about how my personal information or my health information will be used. | Awareness 3 |
| Trusting beliefs | Health-conscious social media companies would be trustworthy in handling my health information. | Trusting beliefs 1 |
|  | Health-conscious social media companies would tell the truth and fulfill promises related to health information provided by me. | Trusting beliefs 2 |
|  | I trust that health-conscious social media companies would keep my best interests in mind when dealing with my health information. | Trusting beliefs 3 |
|  | Health-conscious social media companies are in general predictable and consistent regarding the usage of health information. | Trusting beliefs 4 |
|  | Health-conscious social media companies are always honest with customers when it comes to using health information that I would provide | Trusting beliefs 5 |
| Risk beliefs | In general, it would be risky to give my health information to health-conscious social media companies | Risk beliefs 1 |
|  | There would be high potential for loss associated with giving my health information to health-conscious social media companies. | Risk beliefs 2 |
|  | There would be too much uncertainty associated with giving my health information to health-conscious social media companies. | Risk beliefs 3 |
|  | Providing health-conscious social media companies with my health information would involve many unexpected problems. | Risk beliefs 4 |
|  | I would feel safe giving my health information to health-conscious social media companies. | Risk beliefs 5 |
| Intention | Are you comfortable with providing your own health related information to receive customized pregnant health content via social media? | Intention to reveal health information to receive the health benefit via social media |

**Table S2.** Loadings and Cross-Loadings of the Reflective Items and Internal Consistency Reliability

|  | Collection | Control | Awareness | IUIPC | Trust Beliefs | Risk Beliefs | Intention |
| --- | --- | --- | --- | --- | --- | --- | --- |
| Collection_1 | **0.8688** | 0.2029 | 0.2281 | 0.7228 | -0.3982 | 0.6388 | -0.4193 |
| Collection_2 | **0.851** | 0.2728 | 0.2875 | 0.7493 | -0.4033 | 0.5511 | -0.3089 |
| Collection_3 | **0.9109** | 0.2608 | 0.2607 | 0.7784 | -0.4492 | 0.6172 | -0.3904 |
| Collection_4 | **0.8508** | 0.1831 | 0.2149 | 0.7015 | -0.4233 | 0.6478 | -0.3426 |
| Control_1 | 0.1712 | **0.7468** | 0.3458 | 0.4456 | 0.0301 | 0.0204 | 0.0124 |
| Control_2 | 0.154 | **0.8264** | 0.4856 | 0.5074 | 0.0002 | 0.0547 | -0.1115 |
| Control_3 | 0.2756 | **0.7308** | 0.4242 | 0.5531 | -0.2304 | 0.2701 | -0.1718 |
| Awareness_1 | 0.2041 | 0.4177 | **0.7835** | 0.5269 | -0.1715 | 0.1185 | -0.181 |
| Awareness_2 | 0.175 | 0.4506 | **0.8341** | 0.5345 | -0.1539 | 0.1317 | -0.0416 |
| Awareness_3 | 0.2941 | 0.4392 | **0.7661** | 0.5834 | -0.0495 | 0.2075 | -0.1405 |
| Collection_1 | 0.8688 | 0.2029 | 0.2281 | **0.7228** | -0.3982 | 0.6388 | -0.4193 |
| Collection_2 | 0.851 | 0.2728 | 0.2875 | **0.7493** | -0.4033 | 0.5511 | -0.3089 |
| Collection_3 | 0.9109 | 0.2608 | 0.2607 | **0.7784** | -0.4492 | 0.6172 | -0.3904 |
| Collection_4 | 0.8508 | 0.1831 | 0.2149 | **0.7015** | -0.4233 | 0.6478 | -0.3426 |
| Control_1 | 0.1712 | 0.7468 | 0.3458 | **0.4456** | 0.0301 | 0.0204 | 0.0124 |
| Control_2 | 0.154 | 0.8264 | 0.4856 | **0.5074** | 0.0002 | 0.0547 | -0.1115 |
| Control_3 | 0.2756 | 0.7308 | 0.4242 | **0.5531** | -0.2304 | 0.2701 | -0.1718 |
| Awareness_1 | 0.2041 | 0.4177 | 0.7835 | **0.5269** | -0.1715 | 0.1185 | -0.181 |
| Awareness_2 | 0.175 | 0.4506 | 0.8341 | **0.5345** | -0.1539 | 0.1317 | -0.0416 |
| Awareness_3 | 0.2941 | 0.4392 | 0.7661 | **0.5834** | -0.0495 | 0.2075 | -0.1405 |
| Trust_1 | -0.4611 | -0.0755 | -0.1445 | -0.3843 | **0.8726** | -0.4961 | 0.3826 |
| Trust_2 | -0.4116 | -0.0599 | -0.1262 | -0.34 | **0.8782** | -0.4538 | 0.294 |
| Trust_3 | -0.4368 | -0.0553 | -0.1055 | -0.349 | **0.8894** | -0.4922 | 0.374 |
| Trust_4 | -0.3207 | -0.1096 | -0.1499 | -0.3011 | **0.7654** | -0.4229 | 0.3269 |
| Trust_5 | -0.3939 | -0.121 | -0.1295 | -0.3473 | **0.8224** | -0.4246 | 0.3877 |
| Risk_1 | 0.6798 | 0.2032 | 0.232 | 0.5974 | -0.4176 | **0.8725** | -0.3564 |
| Risk_2 | 0.5627 | 0.0835 | 0.1238 | 0.4471 | -0.3505 | **0.8326** | -0.2853 |
| Risk_3 | 0.5965 | 0.198 | 0.174 | 0.5211 | -0.4206 | **0.8737** | -0.3339 |
| Risk_4 | 0.551 | 0.0719 | 0.0698 | 0.418 | -0.3755 | **0.8098** | -0.2549 |
| Risk_5 | -0.4953 | -0.0874 | -0.1693 | -0.4193 | 0.6109 | **-0.7258** | 0.4692 |
| Intention | -0.4195 | -0.1264 | -0.1529 | -0.3728 | 0.4186 | -0.4253 | 1 |
| Cronbach’s alpha | 0.893 | 0.655 | 0.709 | 0.818 | 0.901 | 0.592 | 1 |
| Dillon-Goldstein’s rho (composite reliability) | 0.926 | 0.812 | 0.837 | 0.858 | 0.927 | 0.816 | 1 |
| Dijkstra-Henseler’s rho (construct reliability) | 0.895 | 0.655 | 0.708 | 0.835 | 0.905 | 0.887 | 1 |

**Table S3.** Discriminant Validity - Squared Interfactor Correlation vs. Average Variance Extracted (AVE)

|  | Collection | Control | Awareness | IUIPC | Trust beliefs | Risk beliefs | Intention |
| --- | --- | --- | --- | --- | --- | --- | --- |
| Collection | 1.000 | 0.070 | 0.081 | 0.720 | 0.231 | 0.495 | 0.176 |
| Control | 0.070 | 1.000 | 0.302 | 0.434 | 0.010 | 0.026 | 0.016 |
| Awareness | 0.081 | 0.302 | 1.000 | 0.479 | 0.024 | 0.038 | 0.023 |
| IUIPC | 0.720 | 0.434 | 0.479 | 1.000 | 0.167 | 0.348 | 0.139 |
| Trust beliefs | 0.231 | 0.010 | 0.024 | 0.167 | 1.000 | 0.294 | 0.175 |
| Risk beliefs | 0.495 | 0.026 | 0.038 | 0.348 | 0.294 | 1.000 | 0.181 |
| Intention | 0.176 | 0.016 | 0.023 | 0.139 | 0.175 | 0.181 | 1.000 |
| AVE | 0.758 | 0.592 | 0.632 | 0.385 | 0.717 | 0.680 | 1.000 |

**Table S4**. Structural Model Multicollinearity Check

| Variable | Collection |  | Control | Awareness | Trust beliefs | Risk beliefs | Intention |
| --- | --- | --- | --- | --- | --- | --- | --- |
| IUIPC | 1.000 |  | 1.000 | 1.000 | 1.000 | 1.200 |  |
| Trust beliefs |  |  |  |  |  | 1.200 | 1.417 |
| Risk beliefs |  |  |  |  |  |  | 1.417 |
